# Electrically connected spin-torque oscillators array for 2.4 GHz WiFi band transmission and energy harvesting

**DOI:** 10.1038/s41467-021-23181-1

**Published:** 2021-05-18

**Authors:** Raghav Sharma, Rahul Mishra, Tung Ngo, Yong-Xin Guo, Shunsuke Fukami, Hideo Sato, Hideo Ohno, Hyunsoo Yang

**Affiliations:** 1 Department of Electrical and Computer Engineering, National University of Singapore, Singapore, Singapore; 2 Centre for Applied Research in Electronics, Indian Institute of Technology Delhi, New Delhi, India; 3 Laboratory for Nanoelectronics and Spintronics, Research Institute of Electrical Communication, Tohoku University, Aoba, Sendai Japan; 4 Center for Science and Innovation in Spintronics, Tohoku University, Aoba, Sendai Japan; 5 Center for Spintronics Research Network, Tohoku University, Aoba, Sendai Japan; 6 Center for Innovative Integrated Electronic Systems, Tohoku University, Sendai, Japan; 7 WPI Advanced Institute for Materials Research, Tohoku University, Aoba, Sendai Japan

**Keywords:** Spintronics, Magnetic devices

## Abstract

The mutual synchronization of spin-torque oscillators (STOs) is critical for communication, energy harvesting and neuromorphic applications. Short range magnetic coupling-based synchronization has spatial restrictions (few µm), whereas the long-range electrical synchronization using vortex STOs has limited frequency responses in hundreds MHz (<500 MHz), restricting them for on-chip GHz-range applications. Here, we demonstrate electrical synchronization of four non-vortex uniformly-magnetized STOs using a single common current source in both parallel and series configurations at 2.4 GHz band, resolving the frequency-area quandary for designing STO based on-chip communication systems. Under injection locking, synchronized STOs demonstrate an excellent time-domain stability and substantially improved phase noise performance. By integrating the electrically connected eight STOs, we demonstrate the battery-free energy-harvesting system by utilizing the wireless radio-frequency energy to power electronic devices such as LEDs. Our results highlight the significance of electrical topology (series vs. parallel) while designing an on-chip STOs system.

## Abstract (web summary)

Spin torque oscillators (STOs) are attractive potential alternative for many high frequency applications, due to their small area and CMOS compatibility. Here, Sharma et al succeed in the electrical synchronization of four STOs, and use their setup to demonstrate wireless and battery-free energy harvesting using eight STOs.

## Introduction

The science of spintronic devices which exploit the spin degree of freedom of electrons is one of the central growing fields in modern science and technology, which already had an enormous technological impact on magnetic sensors and magnetic random-access memories (MRAMs). Spin-torque oscillators^1,2^ (STOs) are a class of emerging spintronic devices that offer a wide range of high-frequency applications^3^ such as a spin-diode rectifier^4,5^, wireless transmission^6^ and reception^7^, digital and analog modulator^6,8,9^, spectrum analyzer^10^, and neuromorphic computing^3,11–13^. STOs offer a nano-sized footprint^14^, complementary metal-oxide-semiconductor (CMOS) compatibility^15,16^, and high-frequency tunability^2,17–19^, as an alternative to the conventional electronic oscillators. Due to the recent efforts of improving the STO output for the potential applications, the power of a single STO has been enhanced up to 10 µW^20^ for the sub-GHz-range single vortex-based STOs, and 2.4–2.8 µW^21,22^ and few hundred nW^23^ for the 1–10 and 10–30 GHz range uniformly magnetized STOs, respectively. Furthermore, the linewidth is scaled down to a few hundreds kHz^24–26^ for the sub-GHz-range single vortex-based STOs. In spite of these few benchmark improvements in the STOs properties, typically a single high-frequency uniform magnetized STO has a low output power (~nW), and a large linewidth (~MHz), making them impractical for real applications. One method to overcome these shortfalls is synchronizing multiple oscillators, as it enhances the output power and reduces the linewidth. Due to their highly non-linear behavior^27,28^ and wideband frequency tunability, STOs can be synchronized by taking advantage of their mutual coupling^12,29–35^.

The mutual coupling of the STOs, which is mediated either via the spin-wave or dipolar coupling^29–33^, is rather limited to a micrometer scale and is generally realized by placing the individual STOs close together. This requirement imposes constraints on designing efficient on-chip STO-based systems and also results in reliability issues. One way to remove such spatial design restrictions is synchronizing the STOs electrically using self-emitted radio frequency (rf) currents. The electrically connected oscillators can be coupled via the inductive/capacitive effects or via the shared ground and source path using a simple electrical topology of parallel and series configurations. Although explored theoretically^36^, the comparison of electrical topology on the performance of STO array has not been demonstrated in experiments, which is an important aspect of any electrically connected network. Till now, a maximum of eight vortex oscillators^35^, which have a non-uniform active magnetic layer has been synchronized^34,35^. The operating frequency of the synchronized vortex oscillators is below 500 MHz due to the weak exchange anisotropy and relatively slow gyration rate of the vortex core as compared to the collective spin precession in a uniform magnetized oscillator^34,35^, which restricts the use of vortex oscillators for high-frequency mobile and wireless applications. In addition, in the current electrical synchronization scheme, the frequency and phase of individual STOs are coupled using separate sources, delay lines^34^, radial combiners^35^, and dedicated attenuators^35^. For instance, the eight vortex oscillators are mutually synchronized using a radial combiner, dedicated permanent magnet, and probing system for individual STO^35^, which complicates the entire process of electrical synchronization and makes it impractical for small on-chip solutions with CMOS technology. While synchronization of a large number of spin Hall nano oscillators (SHNOs) was demonstrated recently^37^, the power scaling of such oscillators is an issue with individual SHNO proving the only pW of the output power.

In this work, we report the electrical synchronization of four non-vortex-based uniformly magnetized STOs arranged in both parallel and series configurations for GHz-range applications. The STOs are synchronized at the highly usable WiFi frequency band of 2.4 GHz, which is crucial for both wireless and computing applications. Unlike previous works in which the STOs are excited by multiple dc sources, we use a single dc source to synchronize canted free layer STOs, thereby simplifying the synchronization scheme. By using parallel and series connections, we highlight the importance of electrical topology by assessing the performance of STO arrays for oscillator and rectification applications. The free-running linewidth of STOs significantly reduces to the range of a few MHz due to mutual synchronization. STOs show an output power in the µW and linewidth in the kHz range when injection-locked with an external rf source. A substantial suppression of the phase noise with four synchronized oscillators is also achieved with rf locking, showing their capability of phase-locking in a large array with good time-domain stability. The synchronized STOs, when used for 2.4 GHz wireless band microwave detection which is an essential feature for energy harvesting, show a large rectified output voltage of 104 mV, a maximum sensitivity of 20,200 mV mW^−1^, and the ac to dc conversion efficiency up to 10%. This performance matrix surpasses the Schottky diode in the sub-microwatt power regime which is useful for nano-sensors in wireless and IoT applications. Using the high rectified voltage of eight oscillators in series, we demonstrate a practical wireless energy-harvesting system capable of powering on a light-emitting diode (LED).

## Results

### Synchronized STOs in parallel and series configurations

**Fig. 1:**
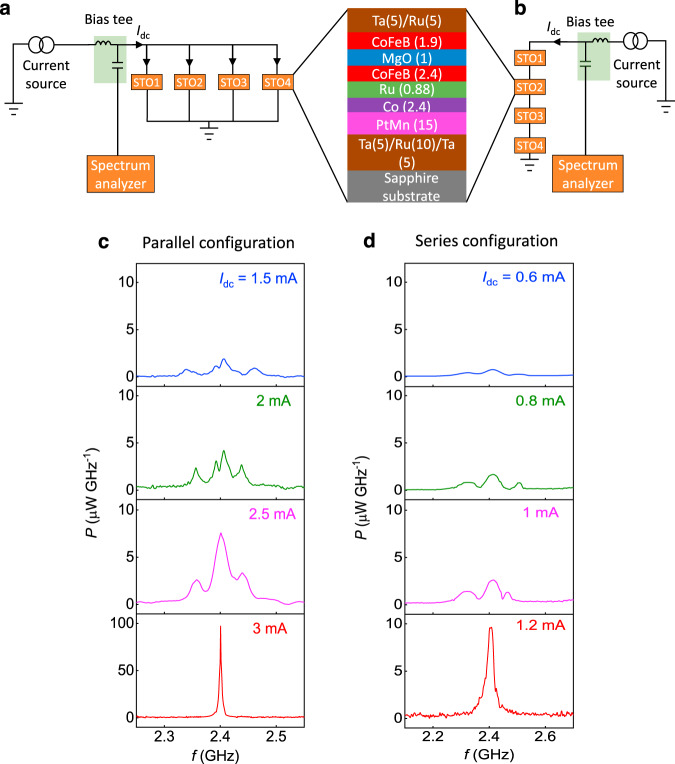
Spin-torque oscillator synchronization with dc bias. **a**, **b** STO measurement set-up using dc bias (*I*_dc_) and spectrum analyzer for the parallel (**a**) and series (**b**) connection, respectively. *I*_dc_ is supplied by the current source and the bias tee isolates the dc bias and the microwave output signal from the STOs. The rf output is then fed into the spectrum analyzer. The device stack is also shown with the numbers in brackets representing the individual layer thickness in nm. **c**, **d** Power spectral density measured in the spectrum analyzer by increasing the dc bias for the parallel (**c**) and series (**d**) connections, respectively. The magnetic field was adjusted with a different dc bias to obtain the single spectral peak at 2.4 GHz. For the condition of synchronization of four oscillators at 2.4 GHz with *I*_dc,sync_ = 3 mA (1.2 mA) for the parallel (series) configuration, the in-plane magnetic field of 180 Oe (140 Oe) is applied along the major axis of the STOs.

For experiments, elliptical (80 × 200 nm^2^) magnetic tunnel junctions (MTJs) with CoFeB as the free layer were used. The thickness of CoFeB was optimized to have a tilted magnetic anisotropy (see film stack in Fig. 1a). These devices show a tunneling magnetoresistance (TMR) of 76–83% and resistance-area product of ~10 Ω µm^2^. These uniformly magnetized oscillators have a large zero-field ferromagnetic resonance (FMR) frequency in the range of 1.95–2.3 GHz by optimizing the canted anisotropy and the exchange coupling values, which can be tuned to the 2.4 GHz WiFi band in the presence of a moderate magnetic field (see the section “Methods”).

Figure 1 shows the experimental setup of STOs for measuring the output power. The STOs are connected in parallel (Fig. 1a) or in series (Fig. 1b) configuration, stimulated by a single dc source. For the parallel connection, at the dc bias (*I*_dc_) value of 1.5 mA and magnetic field (*H*) of 180 Oe, four distinct peaks appear in the power spectrum (Fig. 1c). This corresponds to four independently auto-oscillating STOs. On increasing the *I*_dc_, some of the modes start to merge as shown in Fig. 1c for *I*_dc_ = 2 and 2.5 mA. Finally, at a dc bias (*I*_dc,sync_) of 3 mA, a single spectral peak is observed at 2.4 GHz, signifying synchronization of all four STOs. We denote *I*_dc,sync_ as the current required for the mutual synchronization, which is much higher than the combined threshold current for the auto-oscillations (*I*_th_) of four STOs, where *I*_th_ per STO varies from 0.58 to 0.64 mA. When synchronized, the STOs operate in the mutual synchronization regime, emitting a large microwave current that allows strong STO interaction at a single frequency of 2.4 GHz. The peak output power density in the synchronized state is around 80 µW GHz^−1^, which is 30 times larger compared to that of a single STO. For the series configuration, the STOs move from the unsynchronized to synchronized state as the *I*_dc_ increases from 0.6 to 1.2 mA with *H* = 140 Oe as shown in Fig. 1d. A relatively larger current per STO is required for synchronization in the series configuration compared to the parallel case (current in each parallel oscillator ~*I*_dc_/4), due to the presence of higher spectral noise in the series case which results in linewidth broadening^38^ (discussed later). Hence a higher threshold current is required to minimize the overall linewidth and mutual synchronization of the four oscillators in series. It should be noted that *I*_dc,sync_ is proportional to the number of oscillators that are to be synchronized (Supplementary Note 1).

**Fig. 2:**
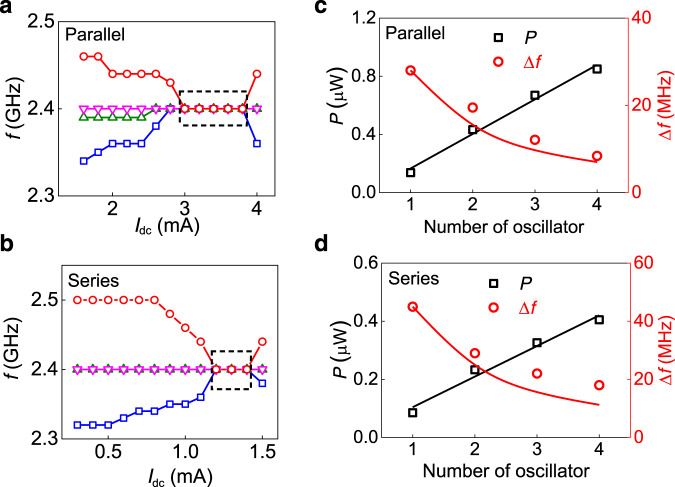
Output frequency and power of the four synchronized STOs. **a**, **b** Evolution of the frequency (*f*) of the four interacting STOs as a function of dc bias (*I*_dc_) for parallel (**a**) and series (**b**) connection. The synchronization condition is indicated by the black dotted boxes. **c**, **d** Maximum power (*P*) and minimum linewidth (Δ*f*) with the different numbers of oscillators (*N*) in parallel (**c**) and series (**d**), respectively. Solid lines are linear fit and *N*^−1^ scaling for the power and linewidth from single to four oscillators, respectively. The *I*_dc_ and magnetic field are adjusted for *N* oscillators for the maximum power and minimum linewidth at 2.4 GHz. For example, for the four synchronized oscillators, the *I*_dc_ and *H* are 3.4 mA (1.3 mA) and 150 Oe (120 Oe) for the parallel (series) configuration, respectively.

The frequencies of the spectral peaks as a function of current for the two configurations are summarized in Fig. 2a, b. The oscillators remain synchronized in the *I*_dc_ range of 3–3.8 mA and 1.2–1.4 mA for the parallel and series connection, respectively. It should be noted that, for the series connection, two of the STOs are synchronized for the measured whole current range as evidenced by the presence of only three spectral peaks in the unsynchronized regime (Fig. 2b). The synchronization of multiple oscillators via microwave current depends on the strength of their coupling, which in turn depends on the emitted power from the individual oscillators. Normally STOs can be mutually synchronized only with a finite difference (Δ*f*_diff_) of their stand-alone oscillation frequency. In contrast to the vortex-based STOs which can be synchronized for a very small Δ*f*_diff_ of a few MHz^34,39^, the STOs in our work can be synchronized for Δ*f*_diff_ as large as 180 MHz due to larger coupling. This feature offers additional flexibility during the on-chip design. The output frequency for both the parallel and series connections is tuned to be ~2.4 GHz in our case, and such electrical STO synchronization in the GHz range has not been reported previously.

The total output power and spectral linewidth are important attributes for an oscillator setup, both of which improve by increasing the number of synchronized STOs (Fig. 2c, d). The maximum output power increases from 0.14 to 0.85 µW (6 times) and the linewidth reduces from 28 to 8.4 MHz (3.3 times), respectively, in the parallel connection of four STOs compared to that of a single oscillator. On the other hand, for a series connection of four oscillators, the maximum output power enhances from 0.089 to 0.4 µW (4.5 times) and the linewidth reduces from 45 to 18 MHz (2.5 times) compared to that of a single oscillator (Supplementary Note 2). The maximum output power and minimum linewidth are observed for the *I*_dc_ slightly higher than *I*_dc,sync_. The enhancement in the power is more than the *N* times (simple addition of individual STO powers when unsynchronized) but less than the ideal achievable value of *N*^2^ in phase-locked oscillators^36^, where *N* is the number of interconnected oscillators. This indicates the phases between individual oscillators are not fully matched and results in a limited enhancement of the power. Furthermore, an increase in the thermal noise and impedance mismatch influences the power enhancement factor by increasing the number of oscillators. In particular, the output power is smaller for the series case due to a larger impedance mismatch of the STO system compared to the 50 Ω load resistance of the spectrum analyzer (Supplementary Note 3). The impedance matching is affected by the stray inductance and capacitance added by the wire bond interconnects. We have found that the inductance in the series configuration and the capacitance in the parallel configuration varies significantly after adding four oscillators. We have found a better impedance match in the parallel configuration due to a trade-off of the stray inductance and capacitance and a relatively less rf power loss than the series configuration. The effect of the stray capacitance and inductance can be controlled in both series and parallel configurations of STOs using hybrid series–parallel combination for better tuning of the impedance, varying interconnection lengths, and introduction of an additional inductance and capacitance in the on-chip circuitry. While the overall output power and the linewidth using the canted free layer-based STOs is relatively poor compared to the synchronized vortex oscillators (µW and kHz range, respectively), the individual vortex oscillator requires a dedicated current source and delay line for the phase and frequency control, which is not scalable and complicates the on-chip design of the synchronized STO system.

### Injection locking for effective synchronization

**Fig. 3:**
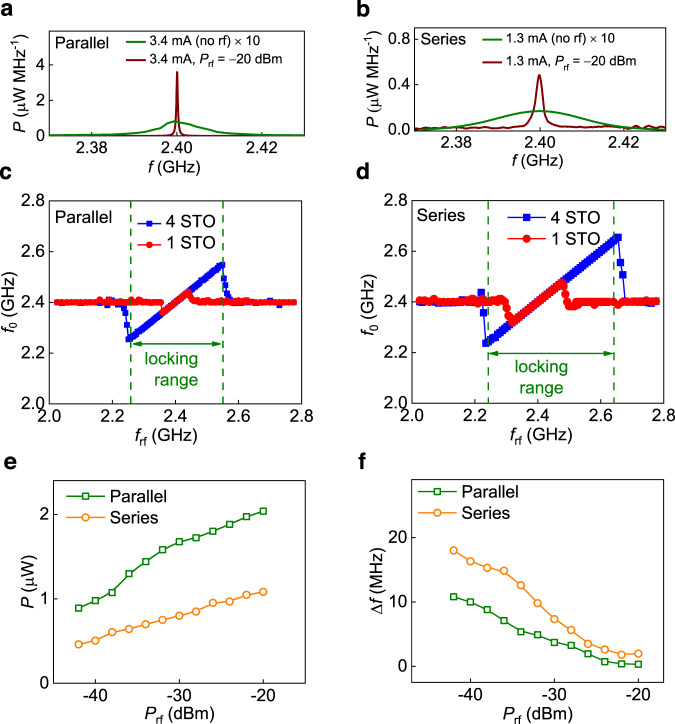
Synchronization with injection locking at 2.4 GHz. **a**, **b** Emitted power spectra of four synchronized oscillators with dc bias only (green) and with both dc bias and rf input (brown) in parallel (**a**) (series (**b**)) configuration at *I*_dc_ = 3.4 mA (1.3 mA) and *H* = 150 Oe (120 Oe), respectively. **c**, **d** STO frequency (*f*_0_) versus radio frequency (*f*_rf_) response for a single oscillator (red) and four oscillators (blue) at *P*_rf_ = −20 dBm for the parallel (**c**) and series (**d**) connection. The locking range (dotted green line) defines the region where *f*_0_ ~ *f*_rf_. **e**, **f** The emitted power and linewidth of synchronized oscillators as a function of applied rf power (*P*_rf_) at *I*_dc_ = 3.4 (1.3 mA) for the parallel (series) configuration, respectively.

We perform injection locking experiments for measuring the response of mutually synchronized STOs to an external rf signal. In this measurement, an rf current (*I*_rf_) in addition to the *I*_dc_ is passed through the coupled oscillators. The frequency of the *I*_rf_ is fixed at 2.4 GHz (Supplementary Note 4). Figure 3a, b shows the comparison of the emitted spectra without (green) and with (brown) *I*_rf_ for the parallel and series connection, respectively. The peak spectral power density of the four synchronized STOs in the presence of *I*_rf_ increases by 44 times to ~3.6 µW MHz^−1^ for the parallel case and to ~0.5 µW MHz^−1^ (30 times) for the series configuration when compared to that with only dc injection (*I*_rf_ = 0).

In the presence of an *I*_rf_, oscillators with a large Δ*f*_diff_ of ~420 MHz can be synchronized due to the increased locking range (*f*_L_) as shown in Fig. 3d. Due to the frequency pulling or frequency locking between oscillators, the overall locking range is enhanced for synchronized STOs as compared to the individual STO (Supplementary Note 5). For example, in the parallel and series configurations, the *f*_L_ is around 300 and 420 MHz, respectively, for an injected rf power (*P*_rf_) of −20 dBm (Fig. 3c,d). These values are ~3.5 times larger compared to that of a single STO. It should be noted that in previous works, very limited *f*_L_ enhancements from 1.69 to 2.87 MHz^39^ and 2–10 MHz^34^ have been demonstrated in a system of two vortexes STOs controlled by multiple dc sources connected in series. The enhancement in the locking range of four synchronized oscillators can be understood by the fact that due to different locking ranges and free-running frequencies of STOs, an STO with a minimum free-running frequency (*f*_0,min_) in the array can lock to the external source frequency (*f*_rf_) at a much lower frequency, *f*_rf_ < *f*_0,min_ and the STO with the maximum free-running frequency (*f*_0,max_) can lock to a much higher frequency, *f*_rf_ > *f*_0,max_. We found that the overall locking range of four oscillators is similar to the merging of the locking range of individual STOs. Once one of the STOs with *f*_0,min_ or *f*_0,max_ locks with the external source frequency, the other oscillators also follow that frequency due to strong interaction. Hence, the overall locking range depends on the free-running frequency and non-linear locking range response of the individual oscillators.

The linewidth and total output power show drastic improvements with injection locking as summarized in Fig. 3e, f. Due to the rf injection locking at *P*_rf_ = −20 dBm, the output power is enhanced from 0.85 to 2.12 µW (2.5 times) in the parallel configuration and from 0.4 to 1.08 µW (2.7 times) in the series configuration compared to the synchronization with the dc bias only (*I*_rf_ = 0). The corresponding reduction in the linewidth is from 8.4 to 0.35 MHz (24 times) in the parallel configuration and from 18 to 1.8 MHz (10 times) in the series configuration. In comparison to a single STO, four injection-locked synchronized oscillators improve the power and linewidth from 0.14 to 2.12 µW (15 times) and from 28 to 0.35 MHz (80 times), respectively, in the parallel configuration. For the series connection, the power is improved from 0.089 to 1.08 µW (12 times), and linewidth decreases from 45 to 1.8 MHz (25 times).

These injection locking results using an external rf source indicate that the STOs may require a coupling strength much higher than the mutual synchronization by the self-emitted rf current to achieve an mW power and kHz linewidth with a larger array of uniformly magnetized STOs. The mutual synchronization can be enhanced between STOs by using individual STOs with high emitting power and minimizing noise in the system for reducing the threshold microwave current required for mutual synchronization. The demonstrated locking range is particularly useful for controlling the transmission and reception of closely spaced rf bands such as 2.4–2.5 GHz using synchronized oscillators. Furthermore, such high-frequency synchronized states of coupled oscillators can be used in fast speed neuromorphic computing^13,40,41^ and Ising computing^42,43^, where an rf source can act as an input and synchronizing states of coupled oscillators as an output. However, for the practical applications, due to the complexity of separating injected rf signal and synchronized STOs output at the same frequency, an ideal requirement is the use of sub-harmonic frequency of the STOs for the electrically injecting microwave current or using the inductive coupling from wireless radiated rf signal at the same frequency.

### Time-domain stability of synchronized oscillators

**Fig. 4:**
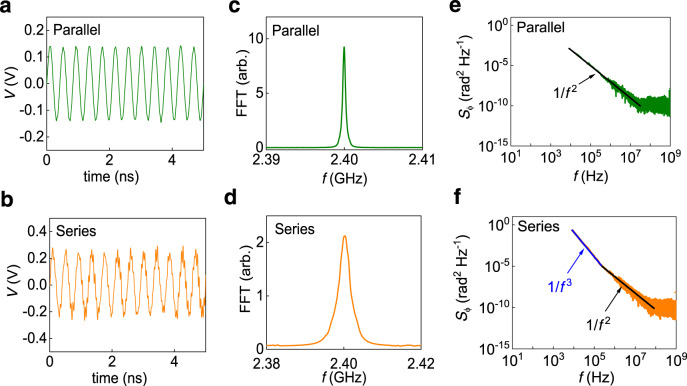
Time-domain measurements and phase noise analysis of injection-locked four synchronized oscillators at *P*_rf_ = −20 dBm. **a**, **b** 5 ns time-domain trace measured in the parallel configuration at *I*_dc_ = 3.4 mA (**a**) and series configuration at *I*_dc_ = 1.3 mA (**b**). **c**, **d** Fast Fourier transform for the 1 ms time-domain trace in parallel (**c**) and series (**d**) at *I*_dc_ = 3.4 mA and 1.3 mA, respectively. **e**, **f** Corresponding phase noise for 1 ms time-domain trace in parallel (**e**) and series (**f**), where the solid black and blue lines in series connections (**f**) shows the fitting corresponding to 1/*f*^2^ and 1/*f*^3^ phase noise, respectively.

Next, we examine the time-domain stability of the injection-locked synchronized STOs. Figure 4a, b shows the 5 ns time-domain trace for the parallel and series configured STOs, respectively. The corresponding fast Fourier transforms (FFT) evaluated using 1 ms trace are shown in Fig. 4c, d. The linewidth measured by the FFT is ~0.38 ± 0.02 MHz (2.06 ± 0.05 MHz) for the parallel (series) connection, while that measured using spectrum analyzer is 0.35 MHz (1.8 MHz). Similar linewidth values from the time-domain and frequency-domain data for the parallel and series connections confirm the reliability of the observed linewidth values.

Phase noise (*S*_ϕ_) measured from the time-domain traces is shown in Fig. 4e, f (Supplementary Note 6). A considerable suppression of phase noise at higher frequency (>30 MHz for parallel configuration and >100 MHz for series configuration) similar to the vortex STOs^25^ is observed (Fig. 4e, f), which suggests all four oscillators follow the phase of the external rf signal overcoming the thermal noise identified by 1/*f*^2^ phase noise for a very short time. Although the suppression of phase noise is considerable and results in a drastic linewidth improvement as shown in Fig. 4e, f in parallel and series configurations, the 1/*f*^2^ phase noise at longer time-segment limits the linewidth improvement. The phase noise in both series and parallel configurations shows a typical 1/*f*^2^ behavior of STOs due to the thermal fluctuations and π-phase slips, which indicates a partial phase locking. The linewidth can be reduced to the source linewidth by injection or sub-harmonic locking as seen in vortex STOs^44^ if 1/*f*^2^ phase noise is completely suppressed^25,45^. The main reason for this limitation is a higher phase noise in uniformly magnetized STOs which might require a much higher rf power for the complete phase noise suppression^25,45^. For the series configuration in Fig. 4f, at a low frequency of <210 kHz, 1/*f*^3^ phase noise (equivalent to 1/*f* frequency noise) is observed. Such a higher-order noise is responsible for time-domain phase fluctuations and hence broadening of the linewidth^38^. This is also an indication of increased mode hopping^46^ in mutually connected multiple STOs in the series connection. Hence, we attribute the time-domain linewidth broadening and instability in the series configuration due to the 1/*f* frequency noise to the short-time desynchronization from the source frequency. The 1/*f* frequency noise is also observed in the unsynchronized individual STOs. Our results suggest that such 1/*f* frequency noise of the individual STOs can be suppressed by a strong mutual synchronization, which we observed only in the parallel configuration.

For quantifying the 1/*f* frequency noise and white noise in broadening the FFT linewidth, we measured the white noise contribution from the mean of Δ*f*_wh_ = *π* × *f*^2^ × *S*_ϕ_ (in the 1/*f*^2^ fitted region of *S*_ϕ_) in Fig. 4e, f^38^. We obtain Δ*f*_wh_ of 1.72 ± 0.09 and 0.36 ± 0.04 MHz for the series and parallel configurations, respectively. The similar values of Δ*f*_wh_ and time-domain FFT linewidth in the parallel configuration reflect the absence of any spurious noise such as the 1/*f* frequency noise^25^. It also substantiates the time-domain stability of synchronized STOs over a long period of time (1 ms). However, a significantly large linewidth compared to the white noise contribution (Δ*f*_wh_) from the time-domain data in the series connection indicates an additional source of noise other than thermal noise^38^.

The time-domain stability of synchronized STOs is determined by the way the STOs are electrically interconnected which is explained as follows. In the parallel configuration, all STOs are directly connected with the source, whereas in the series connection each STO is synchronized through the adjacent one. Due to this difference in the electrical connections, a more reflection loss is observed by adding more STOs in the series connection due to a stray inductance (Supplementary Fig. 3b). This introduces the rf current loss and phase lag in the rf current path from the first to the last STO. On the other hand, the parallel connection shows significantly fewer rf losses and better control of the impedance due to a trade-off of the stray capacitance and stray inductance and hence considerably less phase lag and rf losses by adding more STOs. Additional circuitry, such as electrical delay lines, can help to control the dynamics of individual STOs for the series connection, hence improving their synchronization.

### Enhancement in detection voltage using synchronized oscillators

**Fig. 5:**
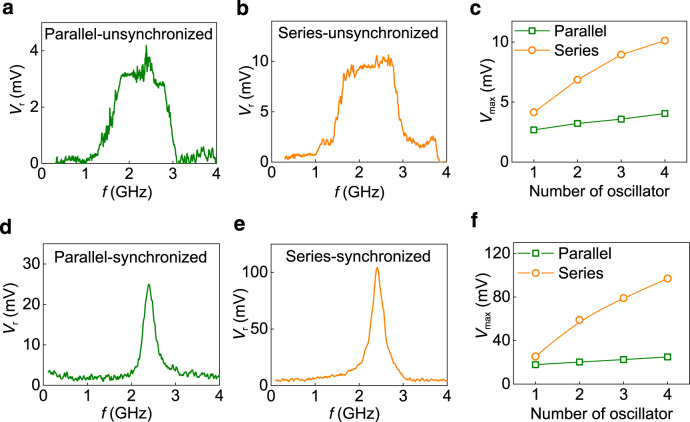
Voltage rectification with four unsynchronized and synchronized oscillators. **a**, **b** Zero dc bias rectification at *P*_rf_ = −10 dBm in parallel at a magnetic field of 180 Oe and series connections at a magnetic field of 150 Oe, respectively. **c** Enhancement in the rectified voltage at 2.4 GHz with increasing the number of oscillators at zero dc bias and *P*_rf_ = −10 dBm. **d**, **e** Rectification results of four synchronized oscillators at *P*_rf_ = −10 dBm in parallel (**d**) at *I*_dc_ = 3.4 mA and *H* = 140 Oe and in series (**e**) at *I*_dc_ = 1.3 mA and *H* = 110 Oe. **f** Enhancement of the rectified voltage at 2.4 GHz with increasing the number of oscillators synchronized with dc bias.

One of the important applications of STOs is in rf-energy harvesting and microwave detection, which utilizes the rectifying property or the spin-diode effect of STOs. The spin-diode effect is demonstrated by measuring the rectified voltage (*V*_r_) using the rf-modulated spin-torque ferromagnetic resonance (RFM-STFMR) technique^4,5^. Due to the canted anisotropy, the free layer of the individual STOs is expected to show a large rf sensitivity as a result of non-linear FMR dynamics^47^. Figure 5a, b shows the rectification results for the unsynchronized condition with zero dc biasing. A significant enhancement in the rectified voltage (*V*_r_) (1.5–3 times) compared to that of individual STOs is observed by increasing the number of interconnected STOs as shown in Fig. 5c. The oscillators show broadband rectification indicated by a plateau in Fig. 5a, b, where the broadband frequency range increases with the rf power (*P*_rf_), similar to previous reports^48,49^. Such a large bandwidth allows STOs for a wideband band-pass detection rather than previously observed low pass broadband detection^48,49^ or high pass broadband rectifier^50^. Overall, the maximum of sensitivity is recorded as ~1850 mV mW^−1^ in the series connection at *P*_rf_ = −35 dBm, which is higher than the zero dc bias sensitivity previously reported using GHz-range MTJ-based spintronic diodes^47,51^. The maximum rectified voltage increases up to ~10.16 mV at *P*_rf_ = −10 dBm for the four oscillators in the series configuration at zero dc bias (Supplementary Notes 7 and 8). The maximum conversion efficiency for unsynchronized four oscillators is ~2% at *P*_rf_ = −25 dBm for the parallel configuration, which is better than any previously reported GHz-range zero dc bias spin-diode efficiency of <1%^47,48,51^. Hence, an array of electrically synchronized STOs is particularly useful for battery-free broadband energy harvesting applications due to the enhanced rectified voltage and detection frequency range.

The linewidth and output voltage of the rectified signal can be improved by synchronizing the four oscillators with *I*_dc_. Figure 5d, e shows *V*_r_ in the presence of a dc bias for these configurations. The *V*_r_ and rf sensitivity is enhanced by mutual locking in the synchronized state. The maximum rectified voltage shows a large improvement of 3 times in the case of series connection as expected due to the addition of *V*_r_ from the individual oscillators (Fig. 5f). The maximum *V*_r_ is found to be ~104 mV in the series configuration, which is sufficiently large for a microwave detector or energy harvesting to run on-chip sensors and higher than previously reported values of ~20–50 mV in spin-diodes^47,50–53^. The parallel connection shows only a slight enhancement of 1.35 times of *V*_r_ when compared to a single oscillator. The maximum sensitivity of the four synchronized oscillators (series configuration) is ~20,200 mV mW^−1^ at 2.4 GHz, showing their suitability for commercial rf detectors with a sensitivity higher than the typical Schottky diode sensitivity of ~2000–5000 mV mW^−1^ ^51,54^ (Supplementary Notes 9 and 10). At *P*_rf_ = −10 dBm, the individual STO shows the conversion efficiency of 1.5–2.25%. The ac to dc conversion efficiency is enhanced to ~10% and 8%, when these oscillators are connected and synchronized in parallel and series, respectively.

### Wireless energy harvesting demonstration using 8 STOs

**Fig. 6:**
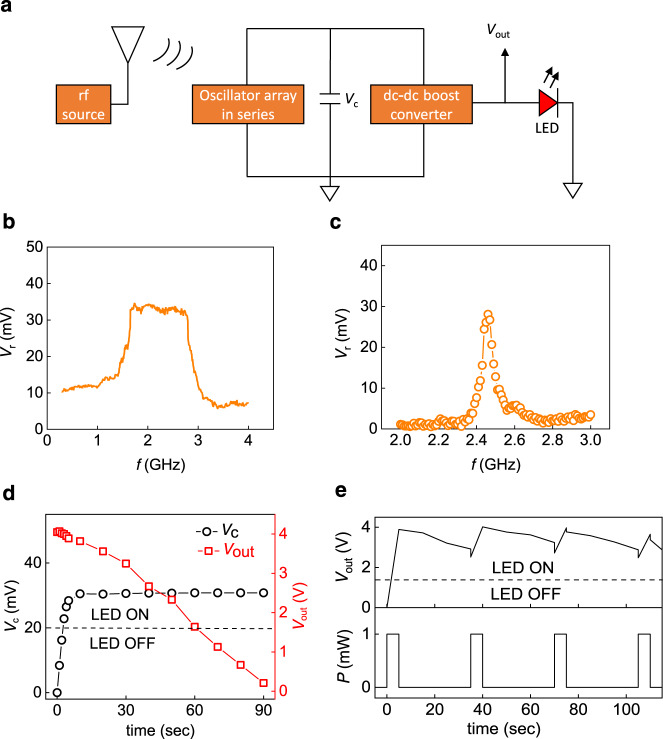
Demonstration of energy harvesting of wireless radio frequency power. **a** Schematic of the circuit used for the energy harvesting. The wireless signal from the horn antenna or microstrip patch-antenna with a power of 0 dBm is irradiated on the array of eight electrically connected STOs in series at zero magnetic field and dc bias. The rectified signal is stored in the capacitor, which is then supplied to the dc–dc boost converter for supplying the voltage to power the red LED. **b** Rectified response of eight oscillators in series when irradiated by a horn antenna fed by a signal generator with the gain of 6–7 dBi in the range of 1–4 GHz. **c** Rectified response of the eight STOs connected in series at rf power of 0 dBm from the patch antenna at zero magnetic field and dc bias. **d** Charging voltage (*V*_c_) of the capacitor from the eight STOs rectified output with time on the left axis. The right axis shows the dc–dc converter output (*V*_out_) when the wireless output is off. **e** The output voltage from the dc–dc boost converter in response to the power of 0 dBm (1 mW) applied from the signal generator to the patch antenna with on and off time of 5 and 30 s, respectively.

Using the advantage of series connection in rectification, we now propose the use of STOs array for practical energy harvesting and microwave detection applications. For this purpose, eight oscillators are connected in series for the broadband frequency response with high rectified voltages. Figure 6a shows the setup for wireless energy harvesting. Figure 6b shows the rectified response of eight oscillators using a horn antenna as a broadband microwave source. The array of eight oscillators generates a dc voltage of more than 5.5 mV from the broadband 0.3–4 GHz frequency range [Fig. 6b], which is omnipresent both indoors and outdoors due to the abundance of WiFi, mobile, and other communication signals. The eight oscillators in series show the maximum rectified voltage of ∼30–34 mV in the range of 1.65–2.8 GHz at *P*_rf_ = 0 dBm and zero dc bias ac to dc conversion efficiency of ∼6% at *P*_rf_ = −20 dBm. This is higher than the reported values for STOs^53,55^ but less than the recent reports of power conversion efficiency of 40% and 40–70% at an input rf power ~0 dBm for MoS_2_-based flexible rectenna and state-of-the-art Si and GaAs rectifiers^56^, respectively. However, these technologies utilize an integrated antenna approach for better impedance matching and maximum power transfer for better conversion. Furthermore, since the input is a wireless rf signal and the output is an additive response of rectified dc voltage, the number of interconnected STOs for energy harvesting is not limited by electrical connection lengths as in the case of mutually synchronized STOs for wireless transmission in the GHz-range. A large array of STOs can be interconnected for achieving much higher efficiency and voltage output targeting state-of-the-art rectifiers and energy harvester capability.

We use a microwave patch antenna as an emitting source for the wireless energy transmission at the resonant frequency of 2.45 GHz, which is rectified by the array of eight STOs in series. The rectified response of the oscillator array without any magnetic field and dc bias is shown in Fig. 6c, which is centered ~2.45 GHz due to the resonant response of the patch antenna. The rectified voltage is stored in the capacitor over time for providing the stable voltage input (*V*_c_) to the dc–dc boost converter for the step-up conversion of ∼30 mV to 3.5–4 V. The left axis of Fig. 6d shows the charging of capacitor via an array of eight oscillators after initiating the transmission through patch antenna. The capacitor takes 3–4 s to charge to *V*_c_ ~ 20 mV, which is the threshold voltage to up-convert the input voltage of 20–400 mV to output voltage of *V*_out_ = 2.5–4.1 V. This up-converted voltage is sufficiently higher than the threshold voltage of 1.6 V required to turn on a LED (Supplementary Note 11). When we stop the wireless signal from the patch antenna, the capacitor allows the output voltage (*V*_out_) from the dc–dc boost converter to discharge slowly. It takes around one minute to reach 1.6 V from 4.1 V as shown in the right axis of Fig. 6d, which enables the LED to be turned on for around one minute even after we switched off the wireless power.

We further demonstrate the capability of this energy-harvesting system in holding dc power using a time-varying signal with 5 s on and 30 s off. Figure 6e shows the applied rf power and corresponding voltage delivered by the energy-harvesting system. Such a demonstration is useful for harvesting energy from a commercialized system such as a WiFi router, where the wireless power is transmitted in modulated data packets that are discontinuous in nature. Our demonstration opens up an avenue for realizing chip-based rf energy-harvesting systems for wireless charging and wireless signal detection.

## Discussion

We have demonstrated electrical synchronization of uniformly magnetized four STOs in parallel and series configurations. The output frequency is tuned at the 2.4 GHz WiFi band, which opens up STO applications in communication, computing, and neuromorphic systems. Furthermore, the oscillation power and linewidth are enhanced with dc and rf inputs. The technological use of STOs can be exploited by their arrangements of the array in parallel and series connections, which offers various applications utilizing the coupled dynamics of multiple oscillators. We have observed mutual synchronization of four STOs which show a power enhancement and linewidth reduction for both series and parallel connections. The major differences observed in the synchronization behavior of STOs in the series and parallel connections are the tuning of impedance and spectral noise, which controls the losses, threshold current for synchronization, and strength of the mutual synchronization defined by the power and linewidth of mutually synchronized oscillators. We find that the parallel configuration is more useful for wireless transmission due to better time-domain stability, spectral noise behavior, and control over impedance mismatch from 50 Ω. On the other hand, series connections have an advantage for rectification due to the additive effect of the diode voltage from STOs. The rectified voltage of 104 mV and 8–10% rectification efficiency are particularly useful in energy harvesting and microwave detector applications. We demonstrate a wireless energy harvester where eight STOs connected in series are integrated as a rectifier at 2.45 GHz to drive conventional electronics such as a light-emitting diode. Our results demonstrate the possibility of using an array of electrically synchronized STOs for on-chip high-frequency GHz-range applications.

## Methods

### Sample preparation

The stack structure of the devices is sapphire substrate/Ta (5)/Ru (10)/Ta (5)/PtMn (15)/Co (2.4)/Ru (0.88)/CoFeB (2.4)/MgO (1)/CoFeB (1.9)/Ta (5)/Ru (5) (thicknesses in nm). MgO is deposited by rf magnetron sputtering, whereas the other layers are deposited by dc magnetron sputtering with Ar gas at room temperature. The base pressure of the deposition chamber is <1 × 10^−6^ Pa. CoFeB (1.9 nm) corresponds to the free layer (FL), where the thickness is designed to have tilted equilibrium anisotropy due to the competition of shape and interfacial anisotropies; the former (latter) favors in-plane (perpendicular) easy axis. CoFeB (2.4 nm) is the reference layer (RL) with in-plane magnetization, and Co/Ru/CoFeB with a synthetic antiferromagnetic (SAF) coupling is exchange biased by the PtMn antiferromagnet. The resistance–area (*RA*) product of the tunnel barrier made of MgO (1 nm) is 10 Ω µm^2^. After the deposition, the stack is annealed at 300 °C for two hours under a magnetic field of 12 kOe applied along the film plane direction to provide the exchange bias. The stack is then processed into elliptical MTJs with the dimensions of 80 × 200 nm^2^ using electron beam lithography and Ar ion beam milling. Subsequently, a co-planar waveguide made of Cr (5 nm)/Au (100 nm) is formed by photolithography and lift-off.

### MTJ characterization

The four STOs are interconnected in an overall length of ~4 mm or less, where the distance between individual STOs is ~1 mm, which allows the microwave current to flow steadily in the four interconnected oscillators without a phase lag by keeping the short wire bonds for minimizing the high-frequency losses and stray inductance and capacitance. For the selection of oscillators in the parallel and series connections, we first determined the dc resistance, tunneling magneto-resistance (TMR), non-linear coefficient (*ν*), and FMR frequency of the neighboring devices. For the synchronization between multiple oscillators, neighboring oscillators with similar resistance, TMR, and FMR frequency values were selected. The FMR frequency was measured using the spin torque (ST)-FMR setup at zero dc bias. The zero bias and zero magnetic field frequency of the devices were found in the range of 1.9–2.3 GHz. Devices showed the frequency per magnetic field tuning of 1.5–2 MHz Oe^−1^. The magnetic field is applied in-plane along the major axis of the STOs. Due to similar FMR frequencies and non-linearity, the frequency tuning in the auto-oscillation region showed a blue shift with 80–120 MHz mA^−1^.

### Oscillator measurements

The synchronization of the multiple oscillators was measured in a spectrum analyzer. The condition of a dc bias and the in-plane magnetic field was adjusted to obtain a single spectral peak in the spectrum analyzer, which is an indication of mutual synchronization. For the injection locking experiment, we used the directional coupler to separate the input of signal of a signal generator from the STOs output signal. Using a directional coupler, we have achieved the isolation of ∼32 dB in the input and output ports. For extracting the output of the synchronized STOs, we first detect the rf signal reflected back from the input port of the directional coupler at *I*_dc_ = 0 mA as the background signal. Next, for extracting the synchronized STOs output shown in Fig. 3a, b, we carefully subtract this rf background from the measured signal at a finite dc bias consisting of the synchronized STOs output signal and rf background. Since the linewidth of reflected rf background is a few Hz only, one can separate it from the STO signal with the linewidth in kHz. In the cases where the rf background subtraction is not accurate, the reflected signal appears as a small single point jump or drop at 2.4 GHz, which we ignored by using appropriate Gaussian fitting of the measured signal. The setup is shown in Supplementary Fig. 4. The oscillation power in the spectrum analyzer is measured in the unit of dBm Hz^−1^, which is converted to nV^2^ Hz^−1^. The signal around STO frequency is integrated over a frequency range to estimate an integrated power in watt. The emitted power (*P*) from the STOs is estimated after correcting from the microwave reflection and transmission losses as measured directly in a vector network analyzer.

### Time-domain measurements

For measuring the time-domain data, we used a 20 GHz and 100 GS s^−1^ high bandwidth oscilloscope (Tektronix DPO72004C). The 1 ms time segment is optimized for taking the reliable phase noise data to match the time-domain FFT linewidth to the spectrum analyzer linewidth within the experimental uncertainty of ±15%. The setup arrangement is the same as shown in Supplementary Fig. 4 by replacing the spectrum analyzer with an oscilloscope. Similar to the spectrum analyzer measurement, the FFT of the time domain data was adjusted to 2.4 GHz using the dc bias and magnetic field near the condition of synchronization observed in the spectrum analyzer data. The time-domain voltage signal is used for the phase noise measurement (Supplementary Note 6).

### Rectification measurements

The rectified voltage was measured using the rf modulated ST-FMR method. The rectified voltage was recorded in a lock-in amplifier, which received the reference frequency of 213 Hz from the local oscillator of the signal generator. The sensitivity in rectification experiments was calculated using the formula *V*_r_/*P*_in_. Here, *V*_r_ is the rectified voltage produced at 2.4 GHz and *P*_in_ is the corrected power, including losses due to the impedance mismatch and the transmission line. The corrected power *P*_in_ was determined by considering the loss from impedance mismatch as measured by a vector network analyzer reflection coefficient from *S*_11_. Similar to the oscillator measurements, we adjust the in-plane magnetic field in the rectification experiment for the maximum rectified voltage at 2.4 GHz. For the ac to dc conversion efficiency, we use *P*_out_/*P*_rf_, where *P*_out_ = *V*_r_^2^/*R*_dc_ is the dc power output and *R*_dc_ is the dc resistance. Since we use a lock-in amplifier for the detection, we ignore any dc component from the current source for the efficiency calculation for synchronized oscillators at a finite dc bias.

### Energy-harvesting demonstration

For the energy harvesting, we used the 2.45 GHz resonant patch antenna with a return loss of more than −35 dB at 2.45 GHz and a gain of 7 dBi. The antenna is fed by the signal generator at 0 dBm. The antenna is placed at ~2.5 cm away from the MTJ array. The rectified voltage from the array of eight connected oscillators is first stored in a 1 F capacitor, which is then supplied to a Linear technology LTC3108 microcontroller chip for the up-conversion of 20–400 mV to 2.35–5 V. When the accumulated rectified voltage from the oscillators reaches the 20–30 mV, the collected power through the capacitor is used for turning on the LED connected at the up-converted output of the microcontroller (see Supplementary Note 11 for more details).

## Supplementary information

Supplementary Information

## Data Availability

The data supporting the findings of this study are available within the paper and other findings of this study are available from the corresponding author upon reasonable request.
