# Supplementary material for: Electrically connected spin-torque oscillators array for 2.4 GHz WiFi band transmission and energy harvesting
Source: Nat Commun. 2021 May 18;12:2924. doi: 10.1038/s41467-021-23181-1 (PMC8131736; doi:10.1038/s41467-021-23181-1)
Supplement: Supplementary file 1 — Supplementary Information [file 41467_2021_23181_MOESM1_ESM.pdf]

Supplementary Information

Electrically connected spin torque oscillators array for 2.4 GHz WiFi band transmission and energy harvesting

Raghav Sharma[1], Rahul Mishra[1,2], Tung Ngo[1], Yong-Xin Guo[1], Shunsuke Fukami[3-7], Hideo Sato[3-6], and Hideo Ohno[3-7] and Hyunsoo Yang[1*]

[1]*Department of Electrical and Computer Engineering, National University of Singapore, 117576, Singapore*

[2]*Centre for Applied Research in Electronics, Indian Institute of Technology Delhi, New Delhi 110016, India*

[3]*Laboratory for Nanoelectronics and Spintronics, Research Institute of Electrical Communication, Tohoku University, 2-1-1 Katahira, Aoba, Sendai 980-8577, Japan*

[4]*Center for Science and Innovation in Spintronics, Tohoku University, 2-1-1 Katahira, Aoba, Sendai 980-8577, Japan*

[5]*Center for Spintronics Research Network, Tohoku University, 2-1-1 Katahira, Aoba, Sendai 980-8577, Japan*

[6]*Center for Innovative Integrated Electronic Systems, Tohoku University, 468-1 Aramaki Aza Aoba, Sendai 980-0845, Japan*

[7]*WPI Advanced Institute for Materials Research, Tohoku University, 2-1-1 Katahira, Aoba, Sendai 980-8577, Japan*

*e-mail: eleyang@nus.edu.sg

Supplementary Note 1. Threshold current for synchronization

The threshold current for the synchronization ($I_{dc,sync}$) depends on the source current and mutual coupling by the microwave signal from the connected oscillators. As mentioned in the main text, we observe a rise in the $I_{dc,sync}$ with additional oscillators. Since in the parallel circuit the total current is divided among oscillators, the threshold current is expected to increase by N times, where N is the number of oscillators. The parallel connection shows an expected 4 times (0.7 to 3 mA) increase in the threshold current for four oscillators as shown in Supplementary Fig. 1. However, series connections show an increase of the threshold current from 0.6 to 1.2 mA for four oscillators. Here, the $I_{dc,sync}$ for the single oscillator is equal to the threshold current for auto-oscillation (I_{th}).

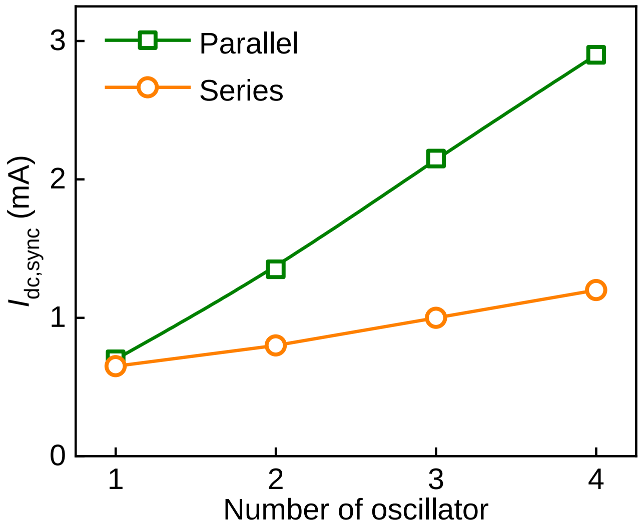

Supplementary Figure 1. The threshold current for synchronization with different number of oscillators in the series and parallel connection.

Supplementary Note 2. Synchronization results from different batches of devices

Synchronized four oscillators show more than N times of the mean power of individual STOs (shown by linear fits in Supplementary Fig. 2 a, c) enhancement in the maximum oscillation power and almost N^{-1} (shown by N^{-1} fits in Supplementary Fig. 2 b, d) reduction in the linewidth in both parallel and series connections, where N is the number of oscillators. The results discussed in the main text are from batch 3 for the parallel connection and batch 5 for the series connection.

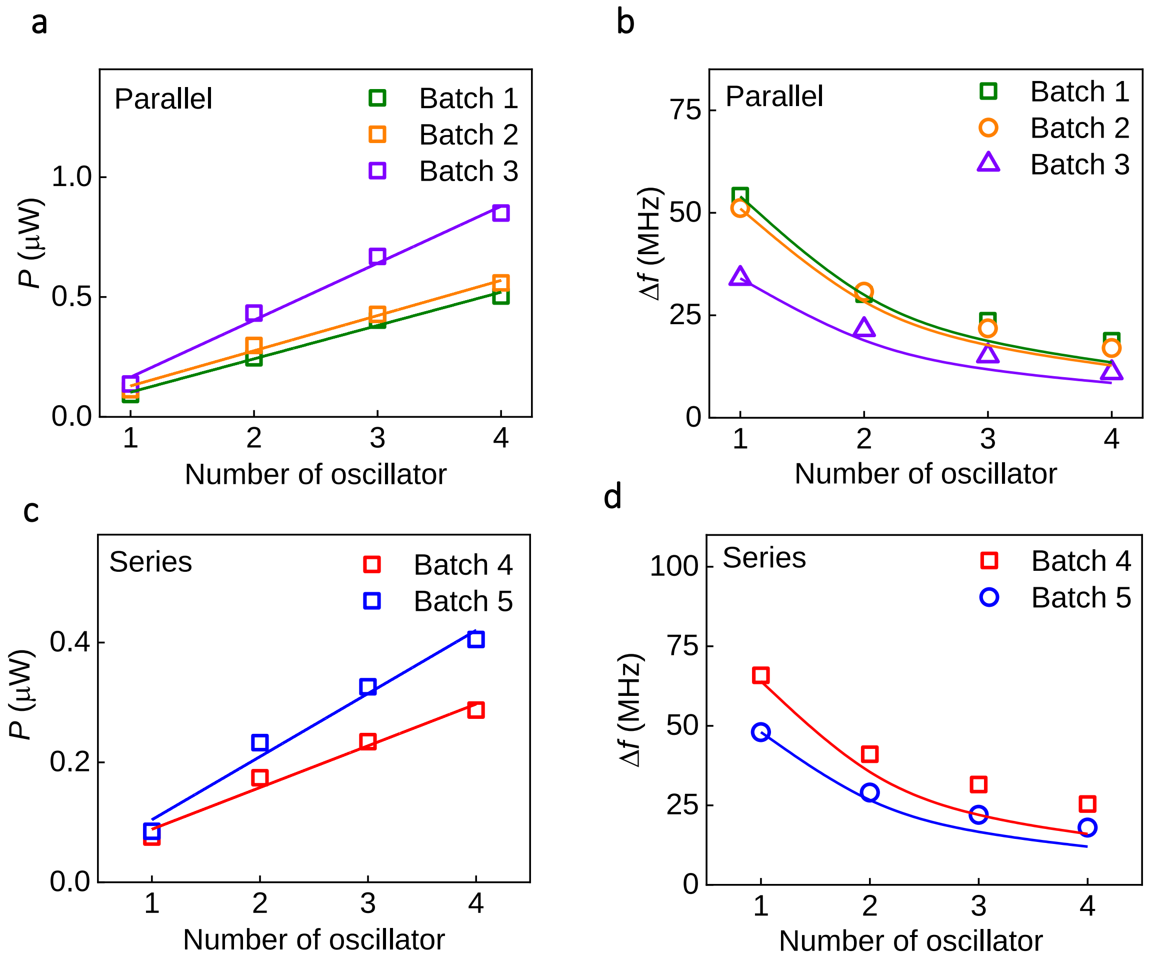

Supplementary Figure 2. Improvement in the maximum oscillation power (*P*) and minimum linewidth (Δ*f*) from different number of synchronized oscillators with dc bias. a, **b** Parallel connection. **c**, **d** Series connection. The open symbols are the data points and solid lines are the linear and N^{-1} scaling for the oscillation power and linewidth from the single STO, respectively.

Supplementary Note 3. Resistance and loss calculation

We measure the resistance using a Keithley 2400 source meter, and impedance mismatch and transmission loss at 2.4 GHz using a S_{11} parameter from a vector network analyzer (VNA). The overall impedance decreases with four STOs in parallel configuration, whereas the impedance (Z) in the series connection increases with 4 connected oscillators (Supplementary Fig. 3a). Similarly, the impedance mismatch loss for the four synchronized oscillators increases in the case of series connection and a slight decrease is observed in the parallel connection as compared to the single oscillator case (Supplementary Fig. 3b). Such a mismatch can be explained by the fact that $Z_{\text{series}} >> 50\ \Omega$ and Z_{parallel} approaching 50 Ω for the four synchronized oscillators. Furthermore, in series connections the effect of stray inductance can be ignored due to the resistance of the MTJs is much higher than the inductor resistance ($2\pi fL$, where f = 2.4 GHz and L = inductance). However, in the parallel connection, the contribution of stray inductance and capacitance becomes significant and plays an important role in impedance matching close to 50 Ω as shown in Supplementary Fig. 3b. With four STOs connected in parallel, the impedance loss approaches to a low value of 0.5 dB. Hence, the impedance mismatch can be tuned using the number of oscillators. The overall loss includes that of components, transmission cables and finite reactance.

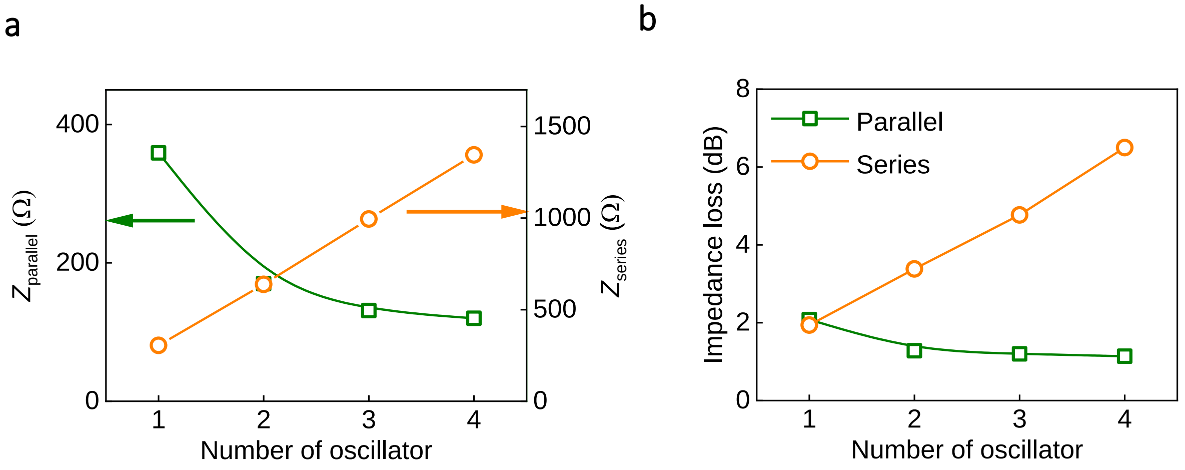

Supplementary Figure 3. The overall resistance and reflection loss. a Parallel and series impedance with increasing the number of oscillators for the results discussed in main text. **b** Impedance loss at 2.4 GHz measured by the S_{11} parameter using a VNA.

Supplementary Note 4. Injection locking experiment set-up

We used the directional coupler to separate the input rf signal of a signal generator from the STOs output signal as shown in Supplementary Fig. 4. The synchronization of the multiple oscillators was measured in a spectrum analyzer.

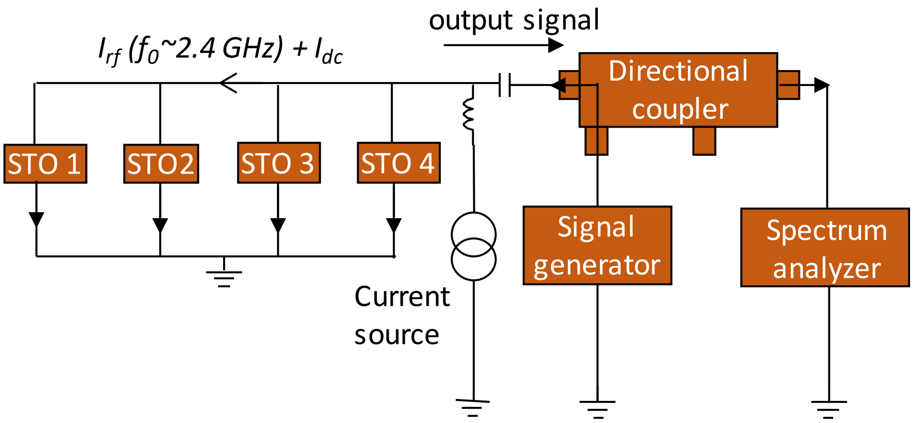

Supplementary Figure 4. Injection locking experiment. Setup for injection locking experiment.

Supplementary Note 5. Enhancement of the locking range through synchronized oscillators

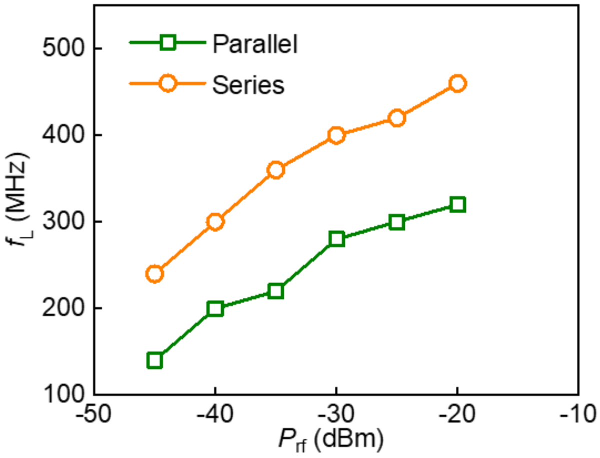

Supplementary Figure 5. Locking range dependence on the input rf power. Locking frequency range (f_L) with varying P_{rf} for four synchronized oscillators in the parallel configuration at I_{dc} = 3.4 mA and series configuration at I_{dc} = 1.3 mA.

Supplementary Note 6. Phase noise measurement from the time-domain trace

For the phase noise measurement, we use the zero-crossover method[1]. The STO voltage can be written as $V(t) = (V_0 + \delta V) \sin [2\pi f_c t + \Delta\phi(t)]$. Here, δV is the voltage fluctuations around the nominal voltage V_0. $\Delta\phi(t)$ is the phase fluctuations and f_c is the crossover frequency. The crossover frequency is calculated using the number of crossover points, where the voltage of STO crosses the zero level, i.e. changes from negative to positive or vice versa. The $\Delta\phi(t)$ is calculated using the relation: $2\pi f_c t + \Delta\phi(t) = \pi/2$. The power spectral density of $\Delta\phi(t)$ [$S_{\Delta\phi(t)}$] is calculated using a hamming window which is used for the phase noise calculation. The phase noise calculation is also verified using the Hilbert transform method[2]. In the Hilbert transform (HT) method, the instantaneous frequency is derived using a complex function $X(t) = V(t) + jHT[V(t)] = A(t)e^{\phi(t)}$. Here, the modulus and argument of the X(t) define the amplitude [A(t)] and the instantaneous phase [$\phi(t)$], respectively. The power spectral density of the instantaneous phase fluctuations, $\Delta\phi(t) = \phi(t) - 2\pi f_0 t$, provides the phase noise, where f_0 is the STO frequency. Both methods yield similar results.

Supplementary Note 7. Zero dc bias sensitivity using unsynchronized oscillators from different batches

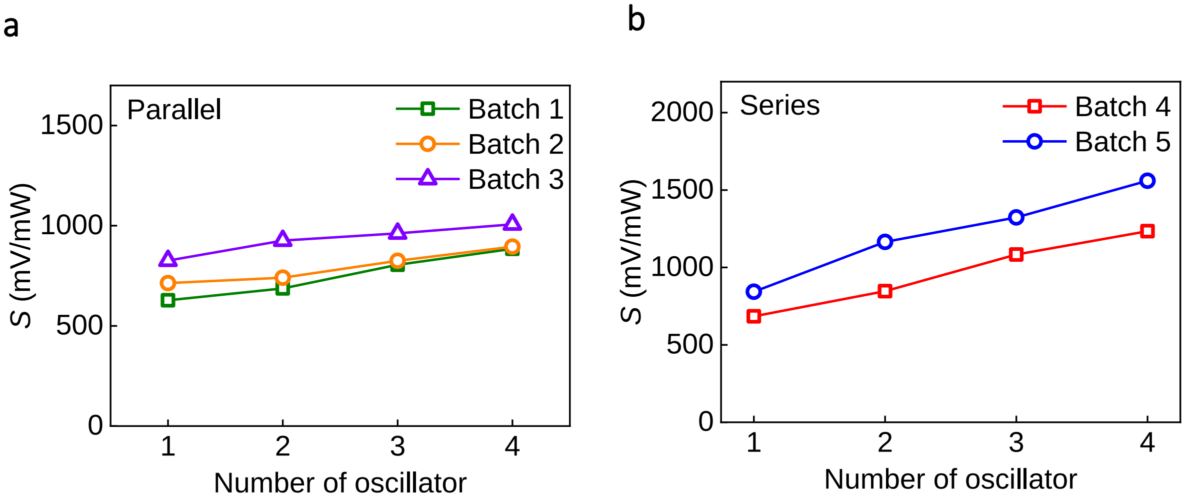

Supplementary Figure 6. Zero dc bias sensitivity from various batches. a, b Enhancement of the rf sensitivity in different batches of four oscillators at zero dc bias and P_{rf} = –35 dBm in the parallel connection (**a**) and series connection (**b**).

Supplementary Note 8. Zero dc bias sensitivity and maximum rectified voltage as a function of rf power for single and four connected oscillators in parallel configuration (batch 3) and series configuration (batch 5)

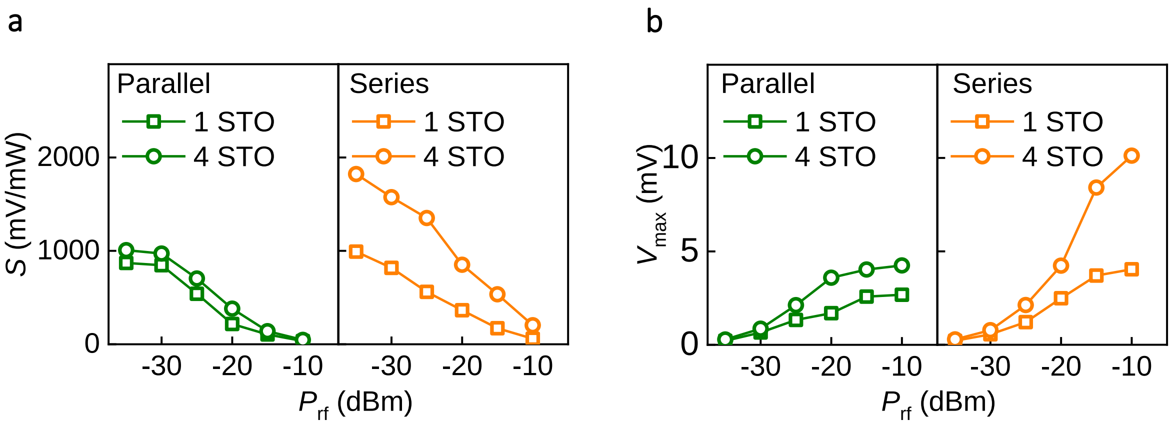

Supplementary Figure 7. Zero dc bias sensitivity and maximum rectified voltage. a Sensitivity as a function of rf power for parallel (green circle) and series (orange circle) connected devices compared with the individual oscillator (square symbol). **b** Corresponding maximum rectified voltage as a function of rf power.

Supplementary Note 9. Sensitivity enhancement using synchronized oscillators with dc bias from different batches

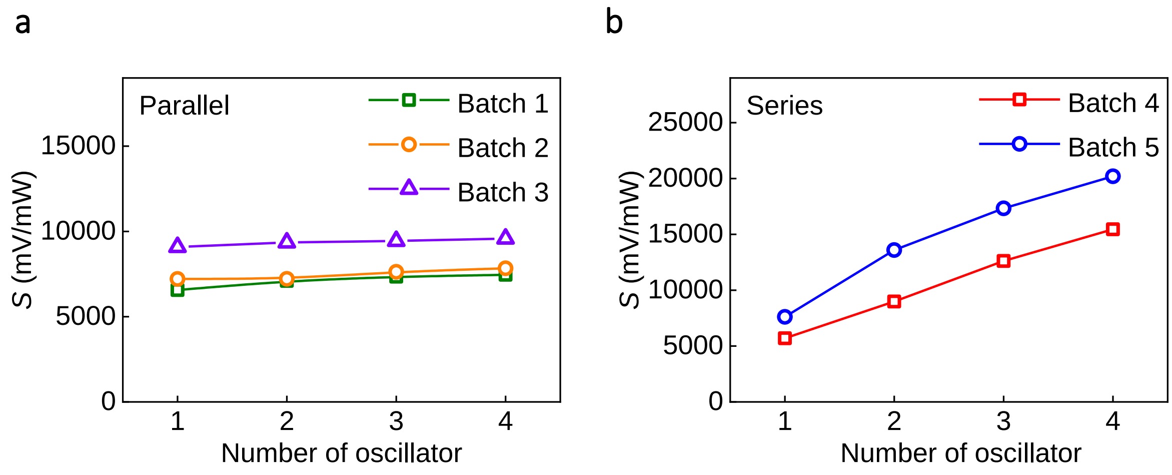

Supplementary Figure 8. Sensitivity with dc bias from various batches. a, b Enhancement of rf sensitivity for four oscillators in different batches at P_{rf} = – 35 dBm in the parallel connection at I_{dc} = 3.4 mA (**a**) and the series connection at I_{dc} = 1.3 mA (**b**).

Supplementary Note 10. Sensitivity and maximum rectified voltage as a function of rf power for single and four synchronized oscillators in parallel configuration (batch 3) and series configuration (batch 5) with dc bias

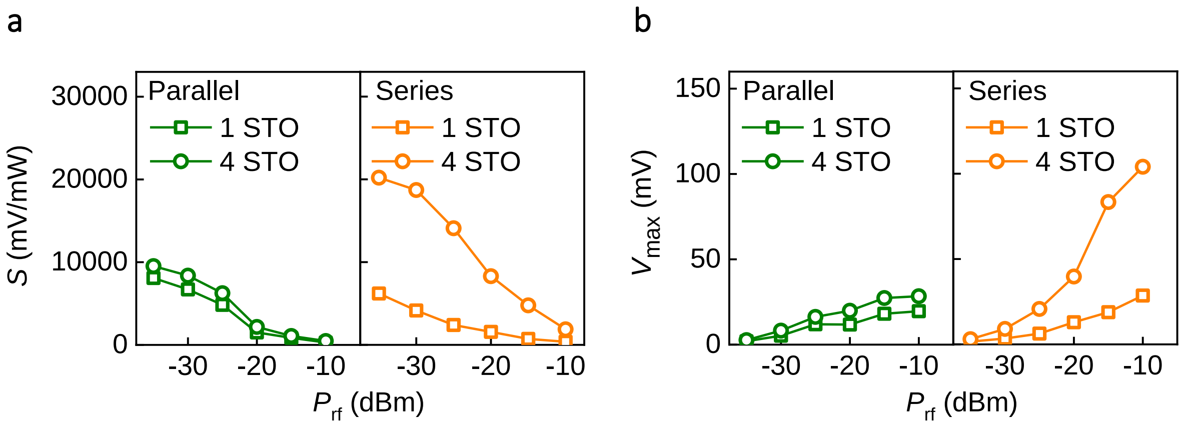

Supplementary Figure 9. Sensitivity and maximum rectified voltage with dc bias. a Sensitivity as a function of rf power for parallel connected devices at I_{dc} = 3.4 mA (green circle) and series connected devices at I_{dc} = 1.3 mA (orange circle) compared to the individual oscillator at I_{dc} = 0.8 mA (square symbol) **b** Corresponding maximum rectified voltage as a function of rf power.

Supplementary Note 11. Demonstration of energy harvesting of wireless rf power for powering electronic devices.

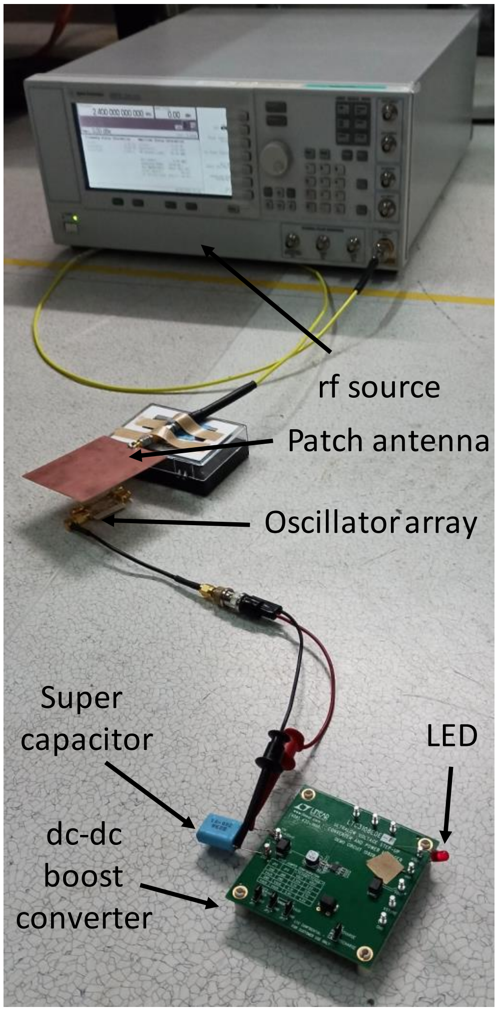

Supplementary Figure 10. Experimental setup for harvesting the 2.4 GHz wireless rf energy to power electronic devices. Demonstration of powering the light emitting diode (LED) by harvesting wireless rf energy using eight oscillators array.

References

1 Keller, M. W., Kos, A. B., Silva, T. J., Rippard, W. H. & Pufall, M. R. Time domain measurement of phase noise in a spin torque oscillator. *Appl. Phys. Lett.* **94**, 193105 (2009).

2 Bianchini, L. *et al.* Direct experimental measurement of phase-amplitude coupling in spin torque oscillators. *Appl. Phys. Lett.* **97**, 032502 (2010).
